# The Crosstalk Between Malignant Cells and Tumor-Promoting Immune Cells Relevant to Immunotherapy in Pancreatic Ductal Adenocarcinoma

**DOI:** 10.3389/fcell.2021.821232

**Published:** 2022-01-11

**Authors:** Xuefei Liu, Ziwei Luo, Xuechen Ren, Zhihang Chen, Xiaoqiong Bao, Jianghua Zheng, Zhixiang Zuo

**Affiliations:** ^1^ State Key Laboratory of Oncology in Southern China, Collaborative Innovation Center for Cancer Medicine, Sun Yat-sen University Cancer Center, Guangzhou, China; ^2^ The Second Clinical Medical School, Lanzhou University, Lanzhou, China; ^3^ Department of Laboratory Medicine, Shanghai University of Medicine and Health Sciences Affiliated Zhoupu Hospital, Shanghai, China

**Keywords:** pancreatic ductal adenocarcinoma, cell-cell communication, immunocyte infiltration, single cell RNA-seq, immunotherapy

## Abstract

**Background:** Pancreatic ductal adenocarcinoma (PDAC) is dominated by an immunosuppressive microenvironment, which makes immune checkpoint blockade (ICB) often non-responsive. Understanding the mechanisms by which PDAC forms an immunosuppressive microenvironment is important for the development of new effective immunotherapy strategies.

**Methods:** This study comprehensively evaluated the cell-cell communications between malignant cells and immune cells by integrative analyses of single-cell RNA sequencing data and bulk RNA sequencing data of PDAC. A Malignant-Immune cell crosstalk (MIT) score was constructed to predict survival and therapy response in PDAC patients. Immunological characteristics, enriched pathways, and mutations were evaluated in high- and low MIT groups.

**Results:** We found that PDAC had high level of immune cell infiltrations, mainly were tumor-promoting immune cells. Frequent communication between malignant cells and tumor-promoting immune cells were observed. 15 ligand-receptor pairs between malignant cells and tumor-promoting immune cells were identified. We selected genes highly expressed on malignant cells to construct a Malignant-Immune Crosstalk (MIT) score. MIT score was positively correlated with tumor-promoting immune infiltrations. PDAC patients with high MIT score usually had a worse response to immune checkpoint blockade (ICB) immunotherapy.

**Conclusion:** The ligand-receptor pairs identified in this study may provide potential targets for the development of new immunotherapy strategy. MIT score was established to measure tumor-promoting immunocyte infiltration. It can serve as a prognostic indicator for long-term survival of PDAC, and a predictor to ICB immunotherapy response.

## Highlights

• We used single-cell RNA-seq to explore the immune microenvironment of PDAC and the crosstalk between malignant cells and immune cells.
• 15 ligand-receptor pairs between malignant cells and tumor-promoting immune cells were identified, which may become new effective immunotherapy target in the treatment of PDAC.
• We constructed a new predictive signature–Malignant-Immune Talk (MIT) that could be used to measure tumor-promoting immune cell infiltration. And high MIT score and MIT-associated mutations usually had a worse response to immune checkpoint blockade (ICB) immunotherapy.

## Background

Pancreatic ductal adenocarcinoma (PDAC) is one of the deadliest human malignancies, which is often diagnosed at advanced stage. The traditional treatment methods such as chemotherapy have limited effects on the patients with advanced PDAC. Developing a new therapy strategy for the treatment of advanced PDAC is urgent.

Cell-cell communications between malignant cells and immune cells frequently occur during cancer progression (Xiong et al., 2019; Deng et al., 2020). Malignant cells will develop abilities to escape the immune surveillance and even educate the immune cells to promote cancer progression. Targeting the ligand-receptor pairs in the malignant-immune cell crosstalk is proved to be effective in many cancers, for example, PD1/PD-L1 is such a pair of ligand-receptor. However, the effect of PD1/PD-L1 immune checkpoint blockade (ICB) in PDAC is still limited (Royal et al., 2010; Zamarin et al., 2020). Therefore, a comprehensive investigation of malignant and immune cell crosstalk in PDAC is needed for finding effective immunotherapy targets.

Recent advances in single cell RNA sequencing (scRNA-seq) allow simultaneous transcriptome-wide quantifications of transcripts in thousands of cells from a biopsy sample, providing opportunities for comprehensively exploring the tumor microenvironment and the crosstalk between malignant cells and immune cells. The previous scRNA-seq studies in PDAC mainly focus on the characteristics and evolution of immune cells and malignant cells. The crosstalk between malignant cells and immune cells is largely neglected.

In the present study, to explore the immune microenvironment of PDAC and the crosstalk between malignant cells and immune cells, we collected public single-cell RNA-seq profiles of 94,910 cells from 24 PDAC samples without any treatment and 11 normal pancreases samples. We revealed a new predictive signature–Malignant-Immune Talk (MIT) that could be used to measure tumor-promoting immune cell infiltration and predict the PD1/PD-L1 ICB immunotherapy response.

## Methods

### Data Acquisition

Single-cell RNA-sequencing data (accession number: CRA001160) of PDAC samples from the initial publication were download and reanalyzed for this manuscript (Peng et al., 2019).

The Cancer Genome Atlas (TCGA) data: Pancreatic cancer RNA Sequencing data (TPM), somatic mutation data and survival information were downloaded from UCSC Xena data portal (https://xenabrowser.net/datapages/). The copy number variation data were calculated by GISTIC2 (Mermel et al., 2011). Data (GSE62452, GSE28735, GSE57495 and GSE85916) with detailed survival data were downloaded from Gene Expression Omnibus (GEO). RNA sequencing of immunotherapy cohort of clear cell renal cell carcinoma (PMID29301960); melanoma (GSE78220, GSE91061 and phs000452) and high-grade glioma (PRJNA482620) was downloaded from the Supplementary Material of the article, GEO and dbgap. RNA sequencing of immunotherapy cohort of bladder cancer was downloaded from R package Mvigor210CoreBiologies (Mariathasan et al., 2018). MSK-IMPACT assay of immunotherapy cohort was downloaded from cbioportal (http://www.cbioportal.org/). Immunohistochemistry (IHC) were downloaded from the Human Protein Atlas database (http://www.proteinatlas.org/). Drug-target genes screened from the Drugbank database (https://www.drugbank.ca/).

### Single Cell Transcriptome Sequencing and Data Preprocessing

The CellRanger software (version 5.0.0) was used for preprocessing of the PE150 Illumina sequencing reads. Briefly, the reads in FASTQ format were aligned to human genome reference (hg38, GRCh38) using STAR (Dobin et al., 2013), and then “cellranger count” was used to derive gene expression matrix for each sample.

Seurat (v3.1.3) R toolkit was used to analyze the single cell transcriptome sequencing data (Butler et al., 2018). Firstly, cells with low quality were filtered out (Wu and Smyth, 2012). Briefly, the dead or dying cells with more than 20% mitochondrial RNA content were removed, and the cells with too low number (less than 200) were also removed. Cell doublets were predicted using DoubletFinder with default parameters. Then, the filtered gene expression matrix for each sample was normalized using “NormalizeData” function in Seurat, and only highly variable genes were remained using “FindVariableFeatures” function in Seurat. Next, “Runharmony” functions in harmony were used to integrate the gene expression matrices of all samples, where batch effects between different samples have been adjusted (Korsunsky et al., 2019). Next, “RunPCA” function was used to perform the principal component analysis (PCA) and “FindNeighbors” function was used to construct a K-nearest-neighbor graph. Next, the most representative principal components (PCs) selected based on PCA were used for clustering analysis with “FindCluster” function to determine different cell types. Lastly, UMAP was used to visualize the different cell types.

We annotated the cell types using the following rules: Based on the most 10 differentially expressed genes that were derived using “wilcoxauc” function in presto, genes such as CD3D, CD3G were used as T cell markers, SDC1, TNFRSF17 were used as plasma cell markers, CD68, CD14 were used as myeloid cell markers, MS4A2, CPA3 were used as mast cell markers, ACTA2, COL6A1 were used as fibroblast/smc cell markers, KRT18, EPCAM were used as epithelial cell markers, VWF, PECAM1 were used as endothelial cell markers, CHGA, CHGB were used as endocrine cell markers, CD19, MS4A1 were used as Bcell markers. CD4, CD8A and CD8B expression were used to differentiate CD4^+^ and CD8^+^ T cells.

Sub-cluster of CD4^+^ Tcell, CD8^+^ Tell, myeloid cell and epithelial cell were named by the first marker gene.

### Pathway/Gene Set Analysis of Single Cell

The pathway/gene set enrichment analysis was performed using the Correlation Adjusted MEan RAnk gene set test (CAMERA)that has been implemented in the SingleSeqGset (version 0.1.2) R package. In brief, the log2 fold change of the mean expression level of a specific gene between the specific cell cluster and the other cells was used as the test statistic. The 50 hallmark gene sets in the MSigDB databases (https://www.gsea-msigdb.org/gsea/msigdb) were used for the CAMERA analysis (Wu and Smyth, 2012).

### Trajectory Analysis

Monocle2 was used to reconstruct single-cell trajectories. Briefly, the “negbinomial.size” function was used to create a “CellDataSet” object from the UMI count matrices with the default setting. The variable genes were defined using the following cutoff: dispersion_empirical > dispersion_fit and mean expression >0.001. Dimensional reduction was performed using the “DDRTree” method, and cell ordering was performed using the “orderCells” function (Qiu et al., 2017).

### Inferring Cell State Transition by RNA Velocity

Spliced and unspliced reads were annotated by velocyto.py used bam and gft files with Default parameters. The loom files were loaded to R using the function “read.loom.matrices.” The calculation od RNA velocity values for each gene in each cell and embedding RNA velocity vector to low-dimension space were done by velocyto.R pipeline. Getting velocyto picture with function “show.velocity.on.embedding.cor” (La Manno et al., 2018).

### DNA Copy Number of scRNA-Seq

DNA copy number variations were detected using inferCNV with default parameters. Cells with more than half of the chromosomes had amplification or loss was defined as malignant cells, others were defined as normal cells (Patel et al., 2014).

Copykat is a powful tools that can delineate copy number and clonal substructure in human tumors from single-cell transcriptome. It can divide cells into aneuploid and diploid automatically with default parameters (Gao et al., 2021).

### Cell-Cell Communication Analysis

To investigate the potential cell-cell communications between immune cells, ductal cells from normal tissue, ductal cells from tumor tissue and malignant cells, we performed ligand-receptor analyses using CellPhoneDB (Efremova et al., 2020) (version 2.0.6). CellPhoneDB applies an algorithm that considers only receptors and ligands with broad expression among the tested cell types, followed by calculating the likelihood of cell-type specificity of a given receptor-ligand complex with enough permutations. We only considered ligands and receptors with expression in more than 20% of the cells in the corresponding sub-clusters to identify the most relevant cell-type specific ligand-receptor interactions. Moreover, we permuted the change of cell type label for each cell at 1,000 times to calculated *p*-value. Finally, we selected the interactions with a *p*-value less than 0.05 and with biologically relevance for further analysis.

### Functional Gene Expression Signatures (Fges) Score Establishment

All knowledge-based functional gene expression signatures (Fges) which were from published literature (Bagaev et al., 2021). Fges scores using MCP_Counter were calculated for all patients in 33 cancer types from TCGA database. Fges score of each cancer is the average of Fges scores of all patients with this cancer type.

### Construction of Malignant-Immune Cell Crosstalk (MIT) Score

We calculated the MIT score according to the following procedure. Firstly, we chose genes highly expressed in malignant cells including ANXA1, C3, CD47, CD55, CD74, CD99, CXADR, FAM3C, LAMP1, LGALS9, LTBR, MDK, MIF and SPP1, which were involved in the crosstalk between malignant cells and tumor-promoting immune cells. These genes were fitted into LASSO Cox regression analysis to select representative makers. This is a logistic regression model that penalizes the absolute size of the coefficients of a regression model based on the value of λ. The most predictive covariates were selected by the minimum (λ min). According to the λ value, each remaining gene would be assigned with a LASSO coefficient, and MIT score was generated using the following formula:

$$MIT\;Score=\overset{n}{\underset{i=1}{\sum }}\left(Expression_{i}*\;LC_{i}\right)$$

where n represents the number of genes, Expression_i_ is the expression level of gene_i_ and LC_i_ is the LASSO coefficient of gene_i_. The R package “glmnet” statistical software (R Foundation) was used to perform the LASSO regression analysis.

### Functional and Pathway Enrichment Analysis of Bulk RNA-Seq

Limma R package was used to identify the genes that were differentially expressed between the low MIT score and high MIT score groups (Ritchie et al., 2015). Signal pathways specificbetween the high and low MIT score groups was explored by GSEA, Gene Set Enrichment Analysis (GSEA) was uesd with adjusted *p*-value < 0.05 using the clusterfiler R package (Yu et al., 2012).

### Immunological Characteristics of the TME in Pancreatic Cancer

To evaluate of immune cell infiltration, the proportions of immune cell types (i.e., TICs) in each samples (with immune infiltration scores) were calculated using the EPIC (Racle et al., 2017) and xcell algorithm (Aran et al., 2017).

### Estimate Drug Sensitivity

The R package “pRRophetic” was applied to estimate the chemotherapeutic and EGFR inhibors responses in the training cohort (Geeleher et al., 2014).

### Statistical Analysis

The Kaplan-Meier method was used to estimate OSthe log-rank test was used to compare the Kaplan-Meier curves. A two-sided *p*-value of less than 0.05 was considered significant. All sample sizes were large enough to ensure proper statistical analysis. Statistical analyses were performed using GraphPad Prism (GraphPad Software, Inc.). *p*-values < 0.05 were considered as statistically significant. All t-test analyses were one-tailed t-tests (paired or unpaired depending on the experiments).

## Results

### Bulk and Single-Cell Gene Expression Analyses Revealed a Tumor-Promoting Immune Microenvironment in PDAC

**FIGURE 1:**
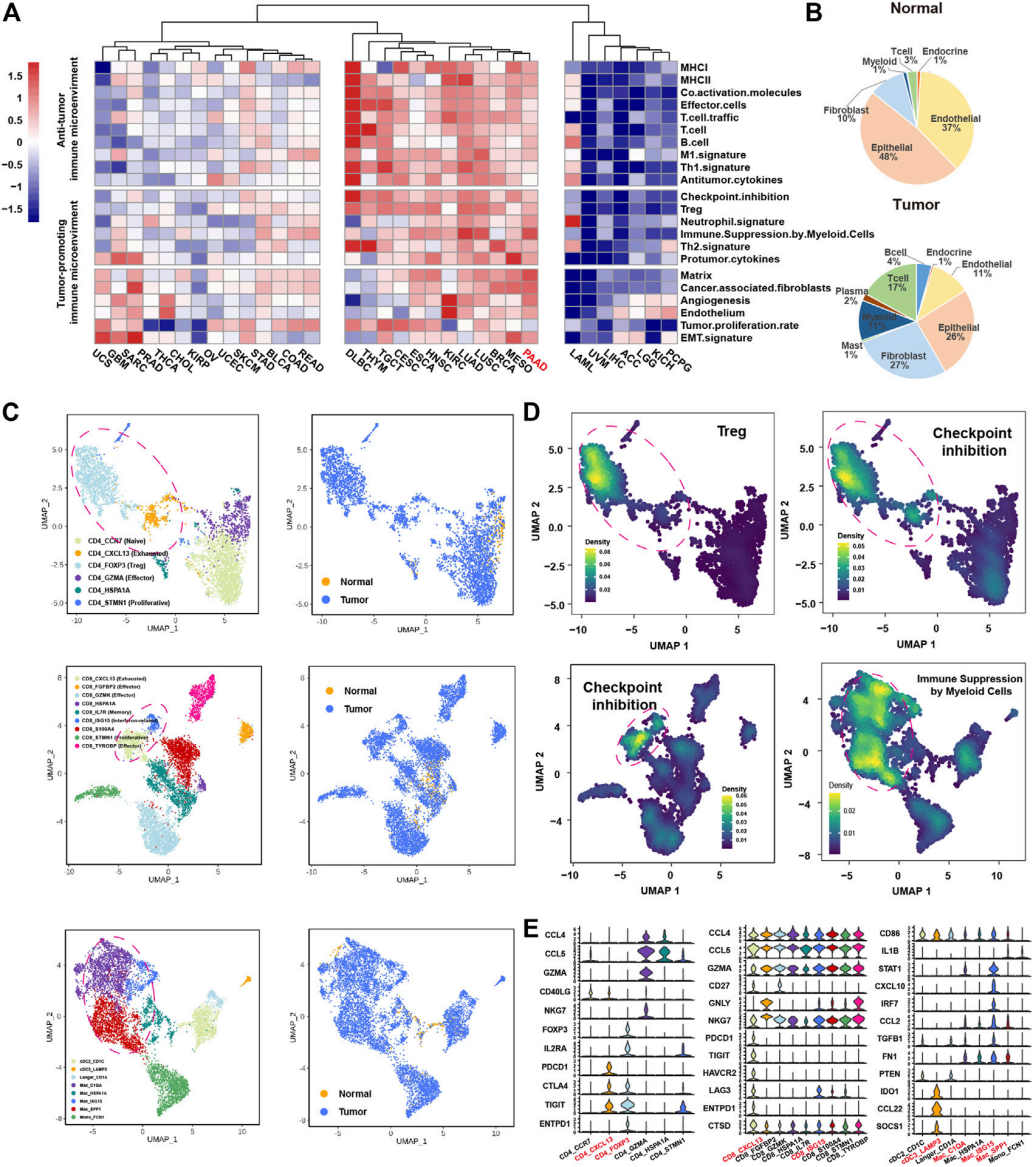
ScRNA-seq and bulk RNA-seq reveals the immune microenvironment in PDAC. **(A)**, heatmap showing the expression of 22 functional gene expression signatures in 33 cancer types from TCGA. **(B)**, pie chart showing the proportion of each cluster for the normal samples (top panel) and tumor samples (bottom panel). **(C)**, Uniform Manifold Approximation and Projection (UMAP) plot of CD4^+^ T cells (top panel), CD8^+^ T cells (middle panel) and myeloid cells (bottom panel), color-coded for subclusters (left panel) and tissue type (right panel), respectively. **(D)**, UMAP plot of CD4^+^ T cells (top panel), CD8^+^ T cells (lower left panel), myeloid cells (lower right panel), color-coded for the average expression of functional gene expression signature. **(E)**, violin plots showing the normalized expression of immune related genes in CD4^+^ T cell (left panel), CD8^+^ T cell (middle panel) and myeloid (right panel) subclusters.

To investigate the immune microenvironment of PDAC, we firstly applied the knowledge-based functional gene expression signatures (Bagaev et al., 2021) (Supplementary Table S1) to the bulk gene expression data of 33 cancer types from TCGA. We identified three groups with different levels of immune infiltration, where PDAC was clustered in the group with high immune infiltrations; however, tumor-promoting immune signatures such as checkpoint inhibition, Treg and immune suppression by myeloid cells were highly enriched in PDAC microenvironment (Figure 1A). Next, we used public single-cell RNA-seq profiles of 111,981 cells from 24 PDAC samples without any treatment and 11 normal pancreases samples to further explore the immune microenvironment of PDAC. After data filtering, doublets removal and batch-effect removal, 94,910 cells were remained, based on which, we obtained nine main clusters including epithelial cells, endocrine cells, endothelial cells, fibroblast cells, mast cells, myeloid cells, plasma cells, T cells and B cells, which is annotated according to the expression of canonical gene markers (Supplementary Figures S1A–C; Supplementary Tables S2, S3). Compared with normal pancreases tissues, the immune cells were greatly expanded in tumor tissues, especially for T cells (3–17%) and myeloid cells (1–11%) (Figure 1B).

We next divided the T cells and myeloid cells into subclusters to further explore the characteristics of these tumor enriched immune cells. The subclusters were named according to known functional markers. The CD4^+^ T cells were divided into six subclusters, including CD4_CCR7, CD4_CXCL13, CD4_FOXP3, CD4_GZMA, CD4_HSPA1A and CD4_STMN1 (Figure 1C, Supplementary Figures S1C–I). The CD8^+^ T cells were divided into nine subclusters, inculding CD8_CXCL13, CD8_FGFBP2, CD8_GZMK, CD8_HSPA1A, CD8_IL7R, CD8_ISG15, CD8_S100A, CD8_STMN1 and CD8_TYROBP (Figure 1C, Supplementary Figures S1D–J). The myeloid cells were divided into eight subclusters, including three dendritic cell clusters (cDC2_CD1C, cDC3_LAMP3, Langer_CD1A) and five macrophage/monocyte cell culsters (Mac_C1Q1, Mac_HSPA1A, Mac_ISG15, Mac_SPP1, and Mono_FCN1) (Figure 1C, Supplementary Figures S1C–I). Consistent with the findings from the analysis of bulk gene expression data, many immune cell subpopulations with tumor promoting immune signatures were presented in PDAC microenvironment (Figure 1D). Specifically, CD4_FOXP3 cells highly expressed “Treg” signature; CD4_FOXP3 cells, CD4_CXCL13 cells, CD8_CXCL13 cells and CD8_ISG15 cells highly expressed “checkpoint inhibition” signature that represents exhausted status (Auslander et al., 2018); Mac_C1QA, Mac_SPP1 and Mac_ISG15 cells highly expressed “immune suppression by myeloid cells” signature (Figure 1D). We further examined the expression of known tumor-promoting markers in these immune subpopulations, confirming that CD4_CXCL13, CD4_FOXP3, CD8_CXCL13, CD8_ISG15, Mac_C1QA, Mac_ISG15 and Mac_SPP1 cells were tumor-promoting immune cells (Figure 1E). Besides, the cDC3_LAMP3 cells that highly expressed LAMP3, IDO1, and CCL21 associated with high maturation and migration ability were also considered as tumor-promoting immune cells (Zhang et al., 2019; Liu et al., 2021) (Figure 1E). Taken together, the above results suggested that immune infiltrations in PDAC tumors might play an important role in promoting PDAC tumor progression.

### Crosstalk Between Malignant Cells and Immune Cells is Associated With Tumor-Promoting Immune Microenvironment

**FIGURE 2:**
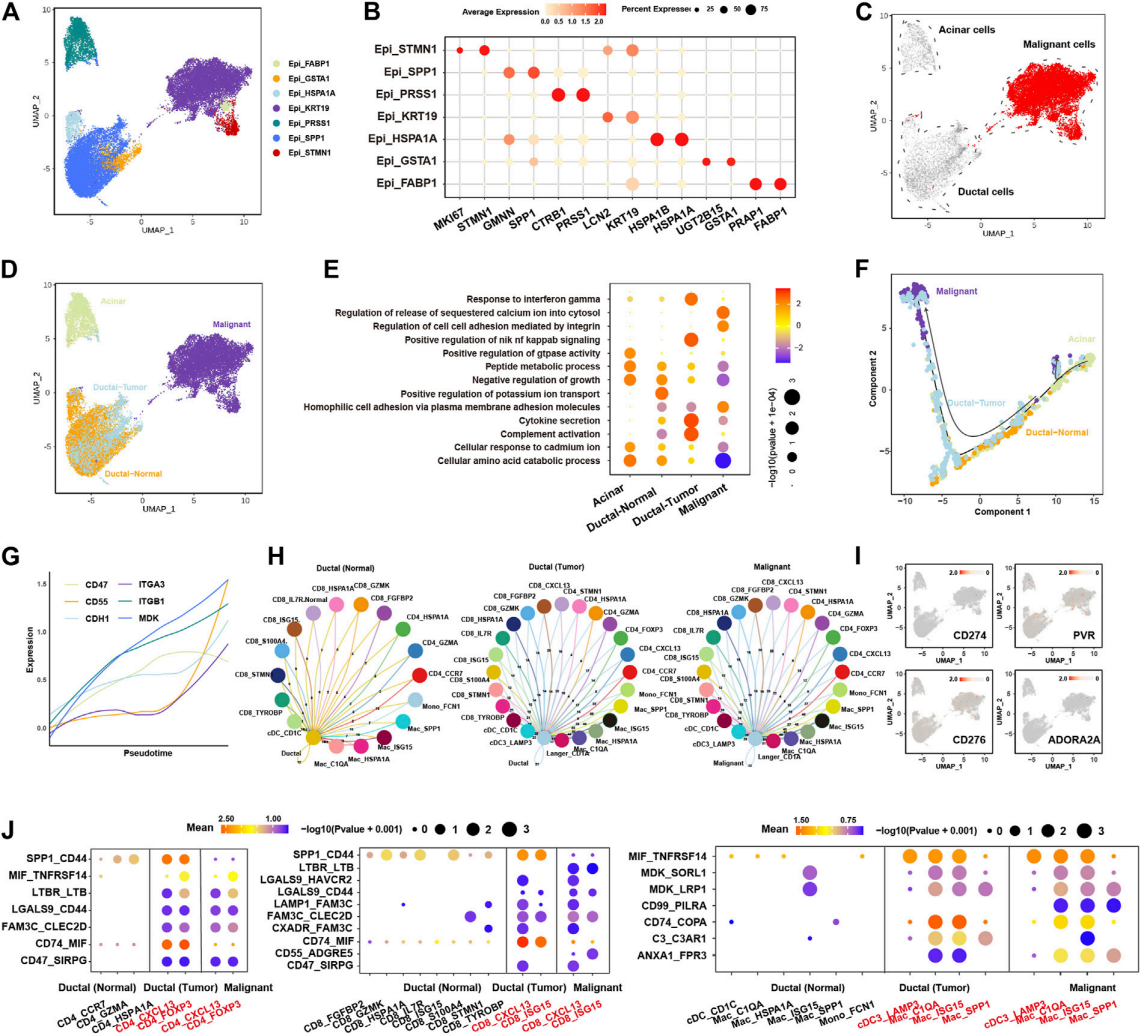
Characteristics and evolution of epithelial cells during cancer progression and their ligand-receptor communications with immune cells in PDAC. **(A)**, UMAP plot of epithelial cells, color-coded for the subclusters of epithelial cells. **(B)**, Dot plot showing the expression of marker genes in each subcluster. **(C)**, UMAP plot of epithelial cells, color-coded for the CNV score calculated by infercnv. **(D)**, UMAP of epithelial cells, color-coded for the four major subclusters: acinar cells, malignant cells, ductal-normal cells and ductal-tumor cells. **(E)**, dot plot showing the enriched pathways in the four major subclusters. The color of each dot represents the normalized enrichment score (NES), while the size of the dot represents *p*-value. **(F)**, trajectory analysis showing the pseudotime of the four major subclusters. **(G)**, line plot showing the expression of cell adhesion molecules (ITGA3, CD47, MDK, ITGB1, CD55, and CDH1) along the pseudotime. Each line with different color represents a gene. **(H)**, circos plot showing the ligand-receptor interactions between immune cells and ductal-normal cells (left panel), ductal-tumor cells (middle panel), and malignant cells (right panel). **(I)**, UMAP of epithelial cells showing the expression of known checkpoint genes (CD274, PVR, CD276 and ADORA2A). **(J)**, the significantly enriched ligand-receptor pairs between in CD4^+^ T cells (left panel)/CD8^+^ T cells (middle panel)/myeloid cells (right panel) and ductal-normal cells/ductal-tumor cells/malignant cells.

Firstly, we clustered the epithelial cells into seven clusters with distinct gene expression pattern (Figures 2A,B, Supplementary Figure S2A). Three epithelial cell clusters (Epi_KRT19, Epi_STMN1, Epi_FABP1) were almost only presented in tumor tissues (Supplementary Figure S2B). By calculating large-scale chromosomal copy number variation (CNV) in each cell type using infercnv, we found that Epi_KRT19, Epi_FABP1, and Epi_STMN1 exhibited remarkably higher CNV compared to other four epithelial clusters (Epi_HSPA1A, EpiGSTA1, Epi_SPP1, Epi_PRSS1) (Supplementary Figure S2C; Supplementary Table S4). Moreover, CopyKAT analysis showed that the cells in Epi_KRT19, Epi_FABP1, and Epi_STMN1 were aneuploidy cells (Supplementary Figure S2D; Supplementary Table S4). Taken together, the cells in Epi_KRT19, Epi_FABP1, and Epi_STMN1 were considered as malignant cells. According to the canonical biomarkers, Epi_PRSS1 was identified as a cluster of acinar cells, and the Epi_HSPA1A, EpiGSTA1, and Epi_SPP1 were identified as clusters of ducal cells. Some cells in cluster Epi_PRSS1 from tumor also exhibited CNV event, which may be related with acinar-ductal metaplasia (ADM). Surprisingly, Epi_HSPA1A, Epi_GSTA1, Epi_SPP1 showed two distinctive CNV states between normal and tumor tissue (Figure 2C, Supplementary Figure S2C), according to which we divided these cells into two clusters, ductal-normal cells from normal tissues and ductal-tumor cells from tumor tissues. Overall, we found four major epithelial clusters in PDAC and normal pancreases tissues, including acinar, malignant, ductal-normal and ductal-tumor (Figure 2D, Supplementary Figure S2E), which have distinct pathway activities, where the ductal-normal cluster exhibits a secretory state, the ductal-tumor cluster exhibits a variety of immune-related functions, and the malignant cluster had a significant enrichment in the cell adhesion pathway (Figure 2E). We found that there is limited composition inter-tumor heterogeneity in four epithelial clusters. As a whole, malignant and ductal-tumor cells predominates in tumor patients, while ductal-normal predominates in normal patients (Supplementary Figure S2G). Interestingly, the distributions of three clusters are different among different stages (Supplementary Figure S2H).

The trajectory analysis suggested acinar cells had the potential to transit to malignant cells, and ductal-normal cells transited to ductal-tumor cells eventually evolved into malignant cells (Figure 2F, Supplementary Figure S3A), consistent with previous report (Ferreira et al., 2017). Cell adhesion molecules such as ITGA3, CD47, MDK, ITGB1, CD55, and CDH1 show a trend of upregulation with pseudotime (Figure 2G), indicating that cell-cell communications play an important role during the evolutionary process of tumor growth and progression. The RNA velocity analysis also supported these evolution paths (Supplementary Figure S3B). We found that the number of receptor-ligand interactions between immune cells and epithelial cells increases during the evolution from normal cells to malignant cells (Figure 2H, Supplementary Table S5), suggesting malignant cells might educate the immune cells into tumor-promoting cells through cell-cell communications.

The most common cell-cell communication molecules such as PD-L1 (CD274) were not expressed in epithelial cells (Figure 2I), consistent with the finding that PD1/PD-L1 ICB has limited effect in the therapy of PDAC. To find new potential checkpoints, we investigated the cell-cell communications between tumor-promoting immune cells (CD4_CXCL13, CD4_FOXP3, CD8_CXCL13, CD8_ISG15, Mac_C1QA, Mac_ISG15, Mac_SPP1 and cDC3_LAMP3) and the ductal epithelial cells (Figure 2J). We found that LGALS9_CD44, MIF_TNFRSF14, CD47_SIRPG, MDK_LRP1 and LGALS9_HAVCR2, CD74_COPA, C3_C3AR1, ANXA1_FPR3 interactions were upregulated in both ductal-tumor and malignant cells versus ductal-normal. FAM3C-related ligand-receptor pairs, like LAMP1_FAM3C, FAM3C_CLEC2D, CXADR_FAM3C, were slightly upregulated in ductal-tumor and malignant cells. Moreover, compared with ductal-normal and ductal-tumor cells, CD55_ADGRE5, CD99_PILRA, ANXA1_FPR3 interactions were drastically upregulated in the malignant cells. SPP1_CD44 interaction was upregulated during the transition from ductal-normal cells to ductal-tumor cells but was downregulated during the malignant transition. Notably, LGALS9_HAVCR2 interaction has been implicated in Treg expansion and cytotoxic T cell apoptosis (Song et al., 2021). These 15 receptor-ligand pairs instead of common checkpoints may lead to high level of tumor-promoting immune cell infiltrations and immune escape. Targeting these receptor-ligand pairs may become new effective immunotherapy strategies in the treatment of PDAC.

### Construction of Malignant-Immune-Talk (MIT) Score to Measure the Tumor-Promoting Immune Microenvironment

**FIGURE 3:**
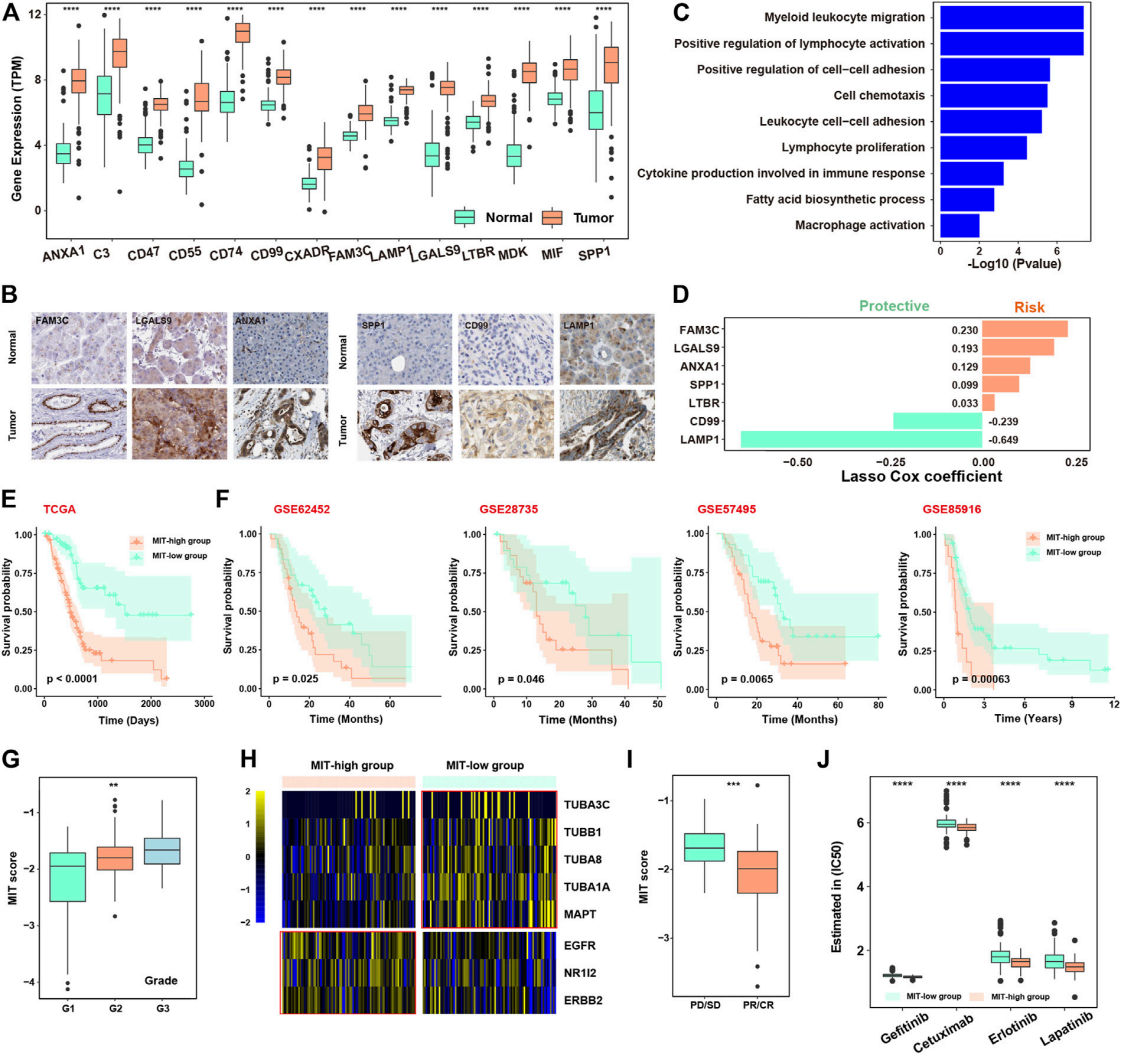
Construction of Malignant-Immune-Talk (MIT) score to measure tumor-promoting immune microenvironment. **(A)**, boxplot showing the differential expression of the 14 ligands from the above 15 ligand-receptor pairs between TCGA tumor and normal samples. *, *p* -value< 0.05; **, *p* -value< 0.01; ***, *p*-value < 0.001 from two-sided paired Student’s t-test. **(B)**, immunohistochemical (IHC) staining of FAM3C, LGASL9, ANXA1, SPP1, CD99 and LAMP1 in normal tissue (top panel) or PDAC tumor tissue (bottom panel). Scale bar, 50 μm. **(C)**, barplot showing the pathways enriched for the 14 ligands from the above 15 ligand-receptor pairs. **(D)**, barplot showing the coefficients of the 7 genes selected from the lasso cox model. **(E)**, Kaplan-Meier curve showing the overall survival difference between patients with high MIT socre and patients with low MIT score in the TCGA PAAD cohort. *p*-value was calculated by the log-rank test. **(F)**, Kaplan-Meier curve showing the overall survival difference between patients with high MIT socre and patients with low MIT score in four independent cohorts of PDAC (GSE62452, GSE28735, GSE57495 and GSE85916). *p*-value was calculated by the log-rank test. **(G)**, box plot showing the MIT scores among different clinical grades. **(H)**, Heat map showing the differential expression of therapy-related genes between high and low MIT groups. **(I)**, box plot showing the different MIT scores between chemotherapy responder group (*p*D/SD) and chemotherapy non-responder group (*p*R/CR). **(J)**, box plot showing the difference in estimated of IC50 for four EGFR inhibitors between high and low MIT group.

We next dedicated to construct a signature to measure the crosstalk between malignant cells and tumor-promoting immune cells. Firstly, the 14 genes (ANXA1, C3, CD47, CD55, CD74, CD99, CXADR, FAM3C, LAMP1, LGALS9, LTBR, MDK, MIF, and SPP1) from the above-mentioned 15 receptor-ligand pairs, which are highly expressed in malignant cells, were considered as potential predictors. We observed all these 14 genes were significantly elevated in tumor tissues than normal tissues in TCGA (Figure 3A), which was confirmed in another cohort (Supplementary Figure S4A). The higher expression of FAM3C, LGALS9, ANXA1, SPP1, CD99 and LAMP1 in tumor tissues compared with normal tissues were confirmed by immunohistochemistry in tumors (Figure 3B, Supplementary Figure S4B). These genes were enriched in immune-related pathways involved in immune cell adhesion, migration and proliferation (Figure 3C). LASSO cox algorithm was used to identify the most robust prognostic genes among the 14 candidate genes and 10-fold cross-validation was applied to overcome the over-fitting effect (Supplementary Figure S4C). As a result, 7 genes (FAM3C, LGALS9, ANXA1, SPP1, LTBR, CD99, and LAMP1) were remained to build an Malignant-Immune-Talk (MIT) score of PDAC using cox regression model (Figure 3D). In the TCGA training set, the 182 patients were divided into two groups according to the MIT score. The patients with higher MIT score had a worse overall prognosis compared to the patients with lower MIT score (Figure 3E, Supplementary Table S6), which was validated in four independent cohorts (Figure 3F), the same trend has been observed in individual genes out of seven genes in MIT score (Supplementary Figure S4D). Multivariate Cox regression analysis revealed that MIT score was the only significant variable for overall survival among various cancer-related hallmarks (*p* < 0.0001; Supplementary Figure S4E). MIT score increased with the progression of the clinical stages and may be used as an indication of early clinical diagnosis (Figure 3G). We found the chemotherapy biomarkers such as TUBB1 and MAPT were highly expressed in MIT-low group and targets of EGFR inhibors such as NR1I2, ERBB2 and EGFR were significantly upregulated in MIT-high group (Figure 3H), suggesting MIT-low group may have a better chemotherapy response, while MIT-high group may respond better to EGFR targeted therapy. Indeed, by analysis of the TCGA PAAD samples with chemotherapy clinical information, we found patients with better chemotherapy response have higher MIT score (Figure 3I). We used pRRophetic package to estimate the drug sensitivity of four common EGFR inhibitors (Gefitinib, Cetuximab, Erlotinib and Lapatinib) in TCGA cohort, and found the estimated IC50 values of these drugs were significantly dropped in MIT-high samples (Figure 3J), confirming that MIT-high group may respond better to EGFR targeted therapy.

### MIT Score is Associated With the Tumor-Promoting Immune Microenvironment

**FIGURE 4:**
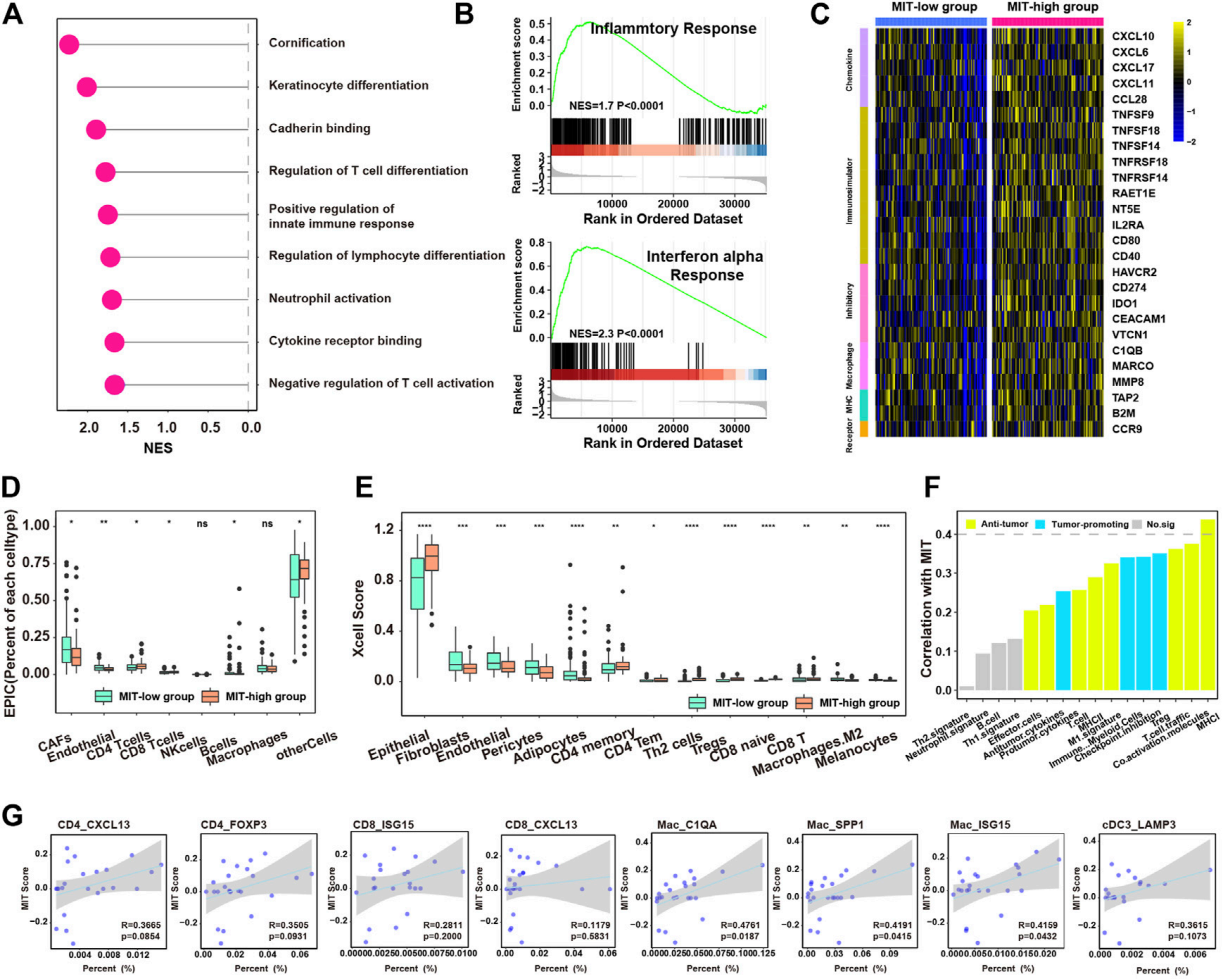
MIT score is associated with tumor-promoting immune microenvironment. **(A)**, enriched pathways for the upregulated genes in MIT high group compared to MIT low group. **(B)**, the two enriched immune hallmark pathways in MIT high group compared to MIT low group derived from GSEA analysis. **(C)**, heatmap showing the differential expression of immune-associated genes between MIT high and low group. The color bar represents the level of expression (Standardized by TPM). **(D)**, boxplot showing the difference in proportions of immune cell types (derived from EPIC algorithm) between MIT high and low groups. *, *p*-value < 0.05; **, *p*-value < 0.01; ***, *p*-value < 0.001 from two-sided paired Student’s t-test. **(E)**, boxplot showing the difference in proportions of immune cell types (derived from xCell algorithm) between MIT high and low groups. *, *p*-value < 0.05; **, *p*-value < 0.01; ***, *p*-value < 0.001 from two-sided paired Student’s t-test. **(F)**, barplot showing the correlation between the MIT score and functional gene expression signatures in PDAC. **(G)**, The correlation between the proportion of tumor-promoting immune clusters (CD4_CXCL13, CD4_FOXP3, CD8_ISG15, CD8_CXCL13, Mac_C1QA, Mac_SPP1, Mac_ISG15 and cDC3_LAMP3) and MIT score.

We next examined whether MIT score represented the tumor-promoting immune microenvironment. We found the 403 genes significantly upregulated in MIT-high samples of the TCGA cohort were enriched in the pathways related to immune-associated tumor promoting such as neutrophil activation and negative regulation of T cell activation (Supplementary Figure S4F, Figure 4A, Supplementary Table S7). GSEA analysis of hallmark pathway revealed that inflammatory response and IFN-a response were enriched in MIT-high group (Figure 4B). A variety of immune-related genes such as chemokines, immunomodulators, inhibitory, MHC molecules, and macrophage-related molecules were also found to be upregulated in MIT-high group (Figure 4C). Notebly, IDO1, the markers of tumor-promoting immune cells cDC3-LAMP3, was upregulated in the MIT-high group. Meanwhile, The TNF superfamily was upregulated in the MIT high group, which may be produced by macrophages, T cells, mast cells, granulocytes, natural killer (NK) cells, and non-hematopoietic cells, all have pro-inflammatory activity and play a key role in various immune and inflammatory processes. MMP8 also was found enrichment in the MIT-high group, which was known to play a role in M2-Macrophage polarization (Wen et al., 2015).

Next, EPIC and xCell algorithms were used to estimate the abundance of various types of cells. In line with the previous results, MIT-high group had higher inflammatory infiltration but tumor-promoting immune environment (e.g., CD8^+^ T, CD4^+^ T cells, Tregs, Th2 and M2 macrophages), while MIT-low group exhibited higher stroma infiltrations (e.g., CAFs, Endothelial and Pericytes) (Figures 4D,E). Meanwhile, the MIT score was positively correlated with most inflammatory and tumor promoting gene expression signature (Figure 4F). By analysis of single-cell RNA sequencing data, MIT score was positively correlated with the proportion of tumor-promoting immune cell clusters, especially the Mac_C1QA, Mac_SPP1 and Mac_ISG15 population (Figure 4G). Taken together, MIT score can indeed be used as an indicator to measure the inflammatory but tumor-promoting immune environment.

### Oncogenic Events Associated With the Crosstalk Between Malignant Cells and Tumor-Promoting Immune Cells

**FIGURE 5:**
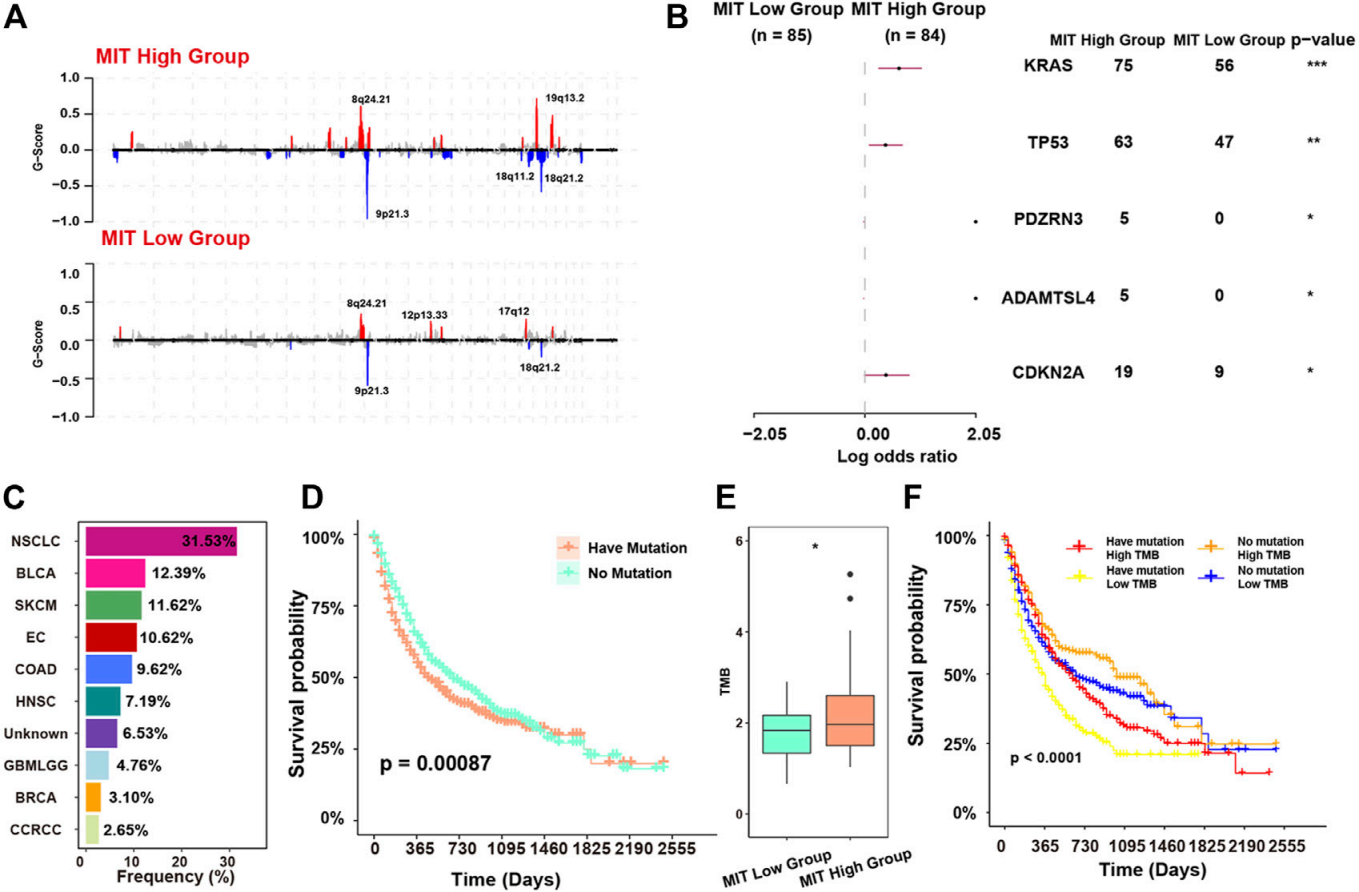
MIT-associated oncogenic events and their relevance to ICB response. **(A)**, recurrent copy number events (GISTIC2 Q < 0.1) in MIT-high group (top panel) and MIT-low group (bottom panel). **(B)**, comparison of mutation rates of individual genes between the MIT high and MIT low groups. *, *p*-value < 0.05; **, *p*-value < 0.01; ***, *p*-value < 0.001 **(C)**, frequency of the mutation of KRAS, TP53, PDZRN3, ADAMTSL4 and CDKN2A in MSK-IMPACT dataset with ICB clinical information. **(D)**, Kaplan-Meier curves showing the survival difference between patients with no mutation and patients having mutation in MSK-IMPACT dataset with ICB treatment. The *p*-value is computed *via* a two-sided log-rank test. Have mutation indicates patients with any of KRAS, TP53, PDZRN3, ADAMTSL4 and CDKN2A mutations. **(E)**, boxplot showing the TMB difference between MIT-high group and MIT-low group. *, *p* -value< 0.05 from two-sided paired Student’s t-test. **(F)**, The Kaplan-Meier curve showing the survival difference among four subgroups of patients. Have mutation representing patients with any of KRAS, TP53, PDZRN3, ADAMTSL4 and CDKN2A mutations. *p*-values were calculated using stratified log-rank test.

We next investigated the oncogenic events associated with the crosstalk (Xiang et al., 2021). For the copy number events, we found 18q11.2 and 19q13.2 were significantly altered in MIT-high group and 12p13.33 and 17q12 were significantly altered in MIT-low group. (Figure 5A). For the somatic mutation events, we found MIT-high group had higher frequency of mutations in KRAS, TP53, CDK2NA, ADAMTSL4, and PDZRN3 than MIT-low group (Figure 5B). This finding is consistent with previous report showing that KRAS mediates crosstalk with the tumor microenvironment, particularly by promoting inflammation and evading the immune response (Liao et al., 2019). CDKN2A has been shown to stimulate cancer immunity (Knudsen et al., 2021). TP53 has been demonstrated to play a role in controlling the crosstalk between malignant cells and other cells (Stein et al., 2019). Interestingly, PDZRN3 and ADAMTSL4 mutations exclusively occurred in MIT-high group, although their occurrences are rare. The potential roles of PDZRN3 and ADAMTSL4 in regulating tumor immune microenvironment are worth exploring. Together, these MIT-associated oncogenic events may drive the crosstalk between malignant cells and tumor-promoting immune cells.

### MIT Score as a Biomarker to Predict the Response to Immune Checkpoint Blockade (ICB)

The MIT-associated mutations (KRAS, TP53, PDZRN3, ADAMTSL4 and CDKN2A) were commonly observed in the samples from a pan-cancer dataset with ICB clinical information and mutation data, and various cancer types including NSCLC, BLCA, SKCM, and EC with the frequencies greater than 10% (Figure 5C). We classified these samples into two groups according to the presence of MIT-associated mutations. We found that the group having mutation had significantly worse survival outcome compared with the group with no mutation (*p* = 0.00087, Figure 5D). The tumor mutational burden (TMB) was known to be associated with high immunogenicity and better ICB response (Schrock et al., 2019). However, we found TMB was significantly elevated in the MIT-high group with poor response to ICB (*p*-value<0.05; Figure 5E). This contradiction is consistent with the immune microenvironment caused by MIT, which is inflammatory but suppressive. Indeed, we found patients with high TMB but having MIT-associated mutations had worse survival than patients with high TMB and no MIT-associated mutations, and patients with low TMB and MIT-associated mutation had the worst survival (Figure 5F).

**FIGURE 6:**
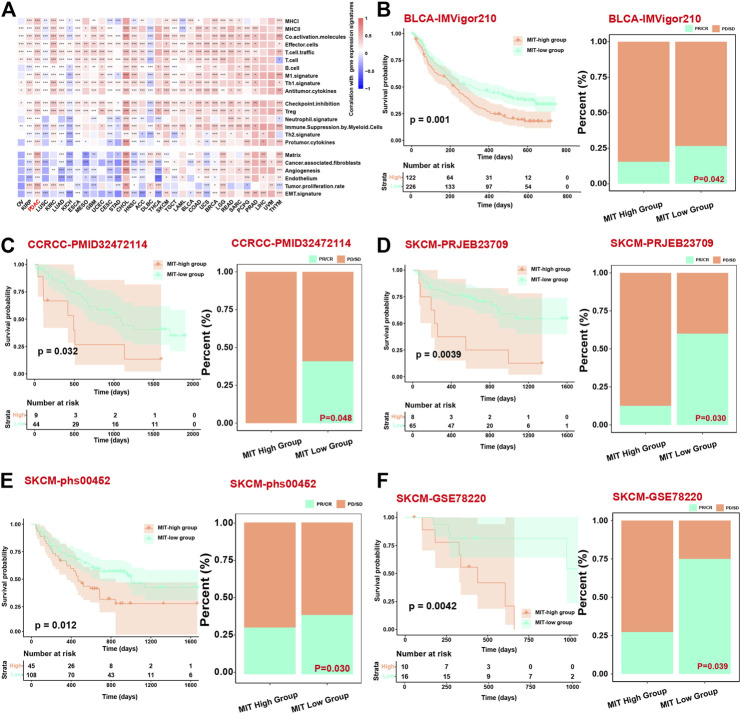
MIT score can be a prognostic factor related to the long-term efficacy of immunotherapy. **(A)**, Heatmap shows the correlation between the MIT score and functional gene expression signatures in 33 cancer types from TCGA. **(B–F)**, Kaplan Meier curves showing the survival difference between MIT-high and MIT-low group (left panel) and bar chart showing the difference in proportion of responders and non-responders between MIT-high and MIT-low group (right panel) in BLCA, CCRCC, SKCM patients, respectively. The *p*-values for survival difference are calculated *via* a two-sided log-rank test, and the *p*-values for proportion difference are calculated from fisher’s exact test.

We next evaluated the association between MIT score and ICB response using the datasets with ICB clinical information and gene expression data. Firstly, we found that the MIT score was significantly positively correlated with the inflammatory and tumor-promoting immune cell gene expression signatures in most of the cancer types in addition to PDAC (Figure 6A), suggesting that MIT score may be used a pancancer biomarker to measure the PD1/PD-L1 independent crosstalk between malignant cell and immune cell. We found in the BLCA (bladder urothelial carcinoma) cohort, patients with high MIT score exhibited worse survival than the patients with low MIT score (*p*-value<0.05); meanwhile, we found more non-responders with ICB were in the MIT-high group (Figure 6B). Similarly, we found that MIT score was significantly associated with a worse response to ICB in other cancer types such as CCRCC (renal clear cell carcinoma) and SKCM (skin cutaneous melanoma) (Figures 6C–F). Together, MIT score and its associated mutations could be used as biomarkers to predict the response to ICB.

## Discussion

In the present study, we comprehensively investigated the immune microenvironment and malignant-immune cell communications in PDAC using both bulk and single cell gene expression data and revealed 15 ligand-receptor pairs that associated with the malignant-immune cell crosstalk. Among these ligand-receptor pairs, several have been reported to play a role in the formation of immunosuppressive microenvironment. For example, the ligand galectin-9 (LGALS9) was reported to negatively regulate T cell responses by promoting CD8^+^ T cell exhaustion and inducing expansion of myeloid-derived suppressor cells *via* the interaction with receptor CD44 (Fujita et al., 2017). Previous studies showed that ANXA1 may have important effects on Treg cell functions (Perretti et al., 2009; Bai et al., 2020). Osteopontin (SPP1) may act as an immune checkpoint to evade immune system independent of CTLA-4/PD-1/PD-L1 (Klement et al., 2018). LTBR and CD99 have also been reported to be closely related to immune regulation (Tang et al., 2016; Takheaw et al., 2019). The ligand-receptor pairs reported in this study may be considered as potential immunotherapy targets for the treatment of PDAC.

Another important contribution of this study is that we proposed a gene expression signature based on ligand-receptor pairs participating in the malignant-immune cell crosstalk, which was named as MIT score. The MIT score can be used to measure the tumor-promoting microenvironment induced by pathways other than PD1/PD-L1 checkpoint inhibition, such as immune suppression by myeloid cells. Patients using PD1/PD-L1 independent mechanism to evade the immune system may not respond to PD1/PD-L1 ICB immunotherapy. Indeed, we found MIT score can be used as a poor survival biomarker in bladder cancer, clear cell renal cell carcinoma and melanoma under ICB immunotherapy.

We also revealed oncogenic events probably driving the malignant-immune cell crosstalk. One of the oncogenic events is KRAS mutations, the occurrence of which was more common in MIT-high patients than MIT-low patients. In solid tumors including PDAC and colorectal cancer, KRAS mutation was reported to induce tumor-promoting TME by inducing regulatory T-cell differentiation or recruiting MDSCs. Meanwhile, the mutant KRAS protein can be released from malignant cells and be further taken by TAMs, leading to an M2-like switch of these TAMs (Zdanov et al., 2016; Liao et al., 2019; Dai et al., 2020). A high level of KRAS activity can produce many factors regulating the maintenance of microenvironment mediators, such as sonic hedgehog, interleukin-6 (IL-6), IL-10, transforming growth factor-β (TGF-β), and prostaglandin E (Charo et al., 2013; Cheng et al., 2019). Another oncogenic event is CDKN2A mutation, which was significantly related to poor T-cell and B-cell infiltration but enriched FOXP3+ Tregs, leading to remarkably shorter survival in patients with PDAC (Wartenberg et al., 2018).

Although crosstalk between malignant cells and tumor-promoting immune cells has been comprehensively investigated in this study, the causality/mechanism has not been explored. Moreover, the predictive value of MIT score for the response to ICB immunotherapy was verified in other cancer types but not in PDAC cohort. Finally, although the MIT was tested in public patient cohorts, a large number of real-world cohorts need to be used for more validation.

## Conclusion

In conclusion, we investigated cell-cell communications between malignant cells and tumor-promoting immune cells in PDAC, providing potential targets for the development of new immunotherapy strategy. Based on these unique crosstalks, MIT score was established to measure tumor-promoting immunocyte infiltration. MIT score can be also used as a biomarker to predict ICB immunotherapy.

## Data Availability

Publicly available datasets were analyzed in this study. This data can be found here: All data used in this work can be acquired from the Gene-Expression Omnibus (GEO; https://www.ncbi.nlm.nih.gov/geo/), The Cancer Genome Atlas (TCGA) data portal (https://portal.gdc.cancer.gov/), The European Molecular Biology Laboratory (https://www.embl.org/), and the Human Protein Atlas (HPA, https://www.proteinatlas.org/).

## Abbreviations

PDAC, pancreatic ductal adenocarcinoma; ICB, immune checkpoint blockade; PD-1, programmed cell death 1; PD-L1, programmed death-ligand 1; scRNA-seq, single cell RNA sequencing; MIT, malignant-Immune Talk; TCGA, the cancer genome atlas; GEO, gene expression omnibus; IHC, immunohistochemistry; GRCh38, human genome reference; ADM, acinar-ductal metaplasia; Tregs, regulatory T cells; NK, natural killer cells; Th2, T help 2 cells; TMB, tumor mutational burden; CCRCC, renal clear cell carcinoma; SKCM, skin cutaneous melanoma.
