# Supplementary figures and images for: The Crosstalk Between Malignant Cells and Tumor-Promoting Immune Cells Relevant to Immunotherapy in Pancreatic Ductal Adenocarcinoma
Source: Front Cell Dev Biol. 2022 Jan 11;9:821232. doi: 10.3389/fcell.2021.821232 (PMC8787220; doi:10.3389/fcell.2021.821232)

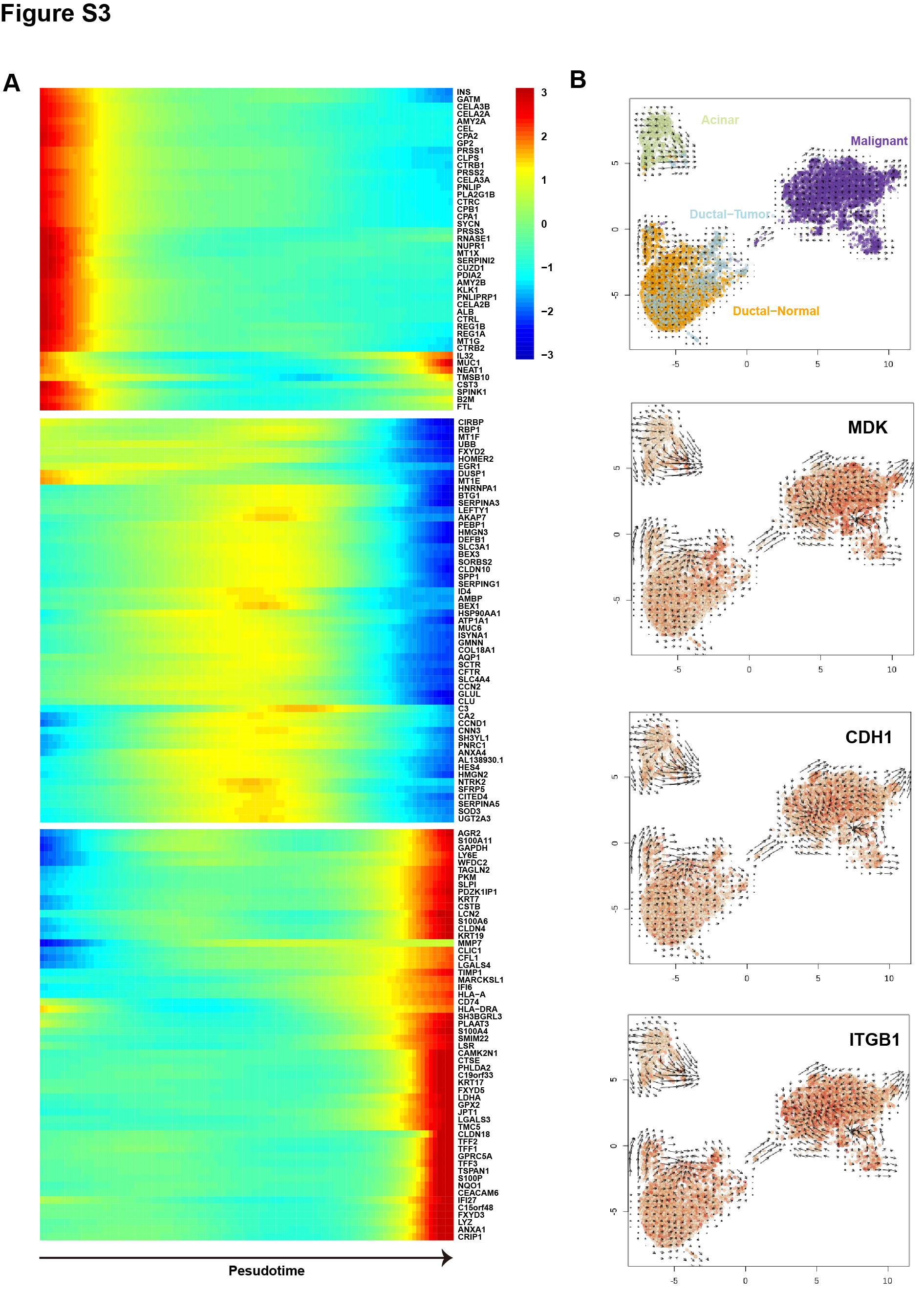

Supplement: Supplementary file 2 [file Image3.JPEG]

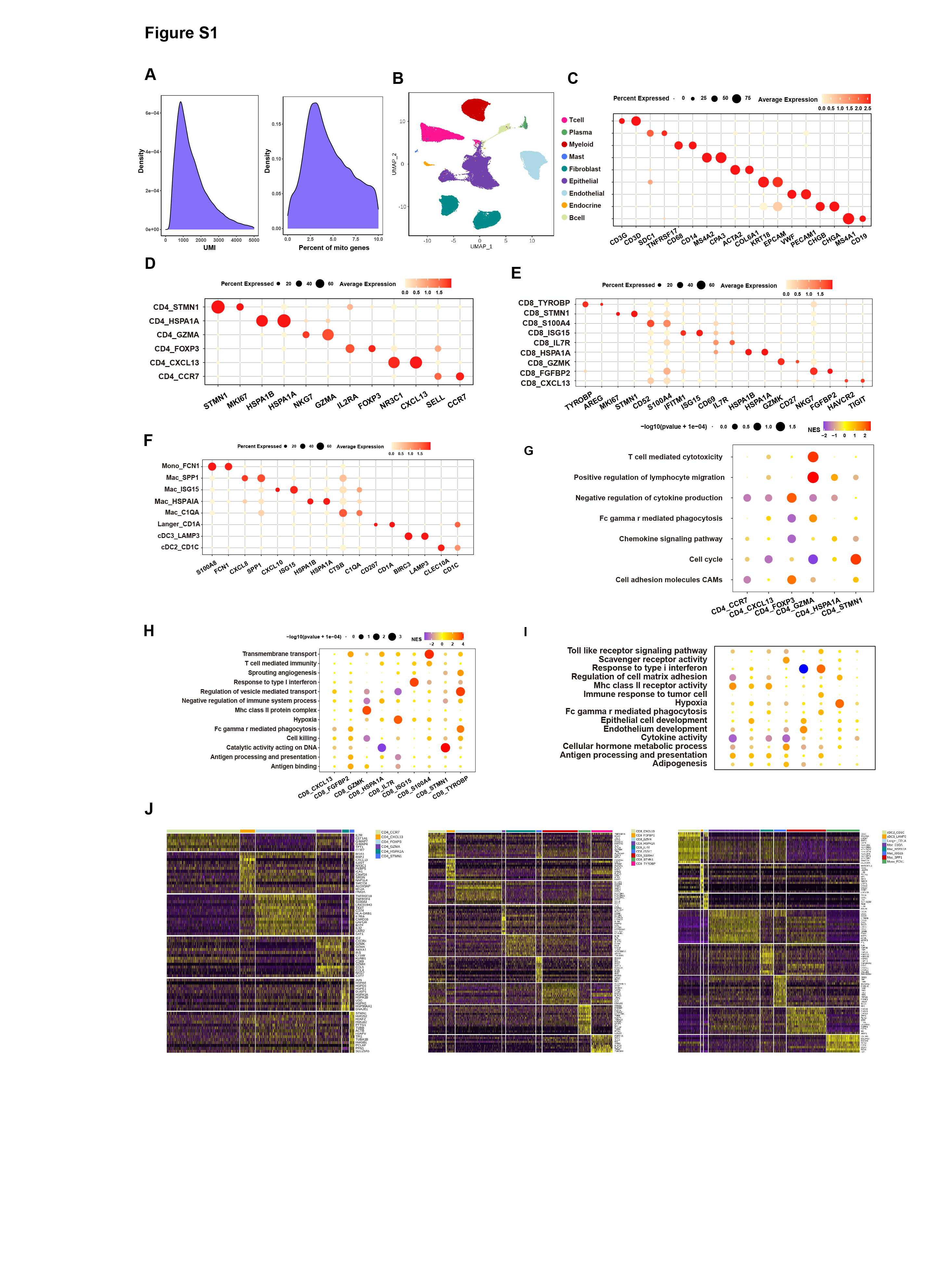

Supplement: Supplementary file 4 [file Image1.JPEG]

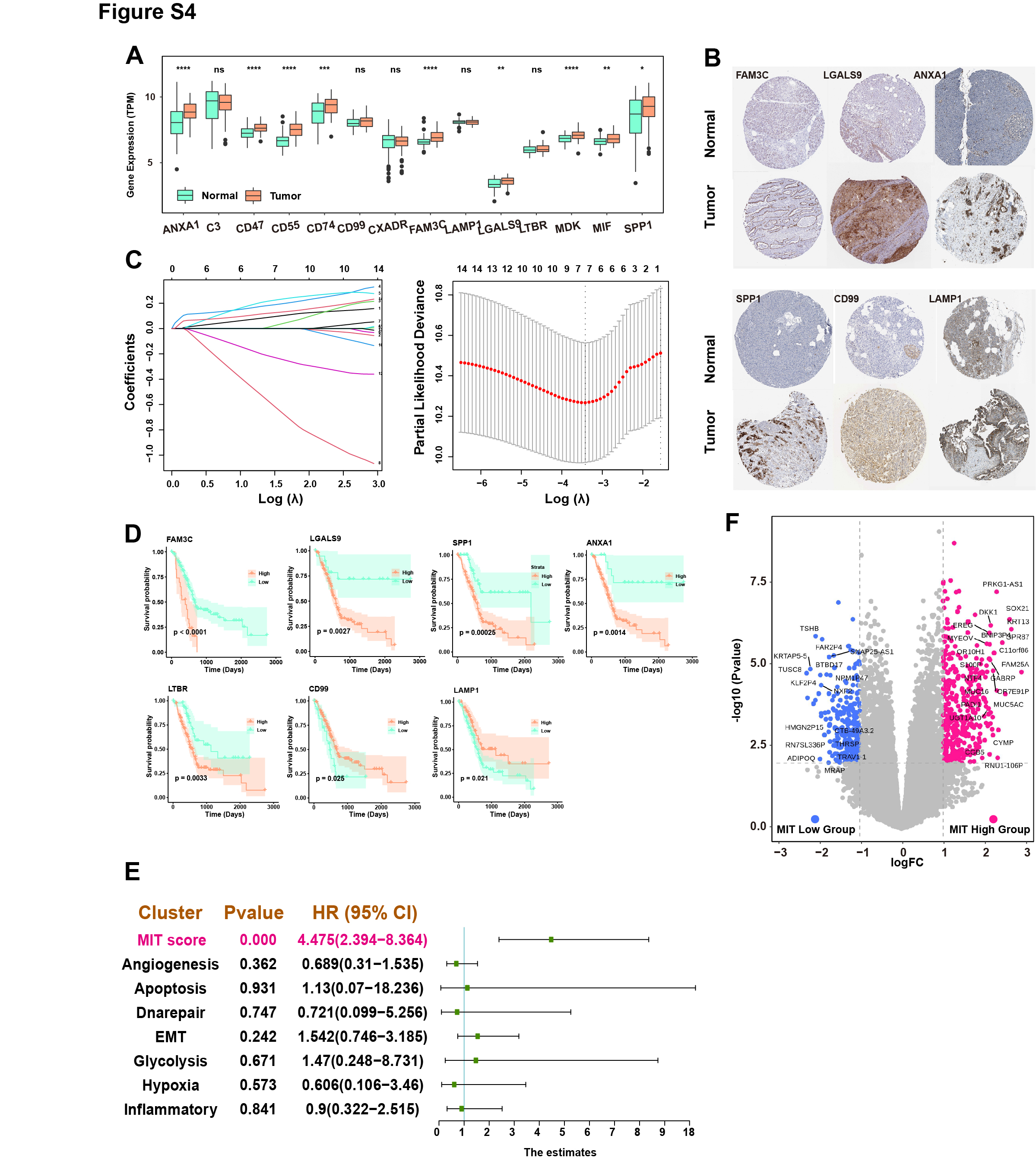

Supplement: Supplementary file 5 [file Image4.JPEG]

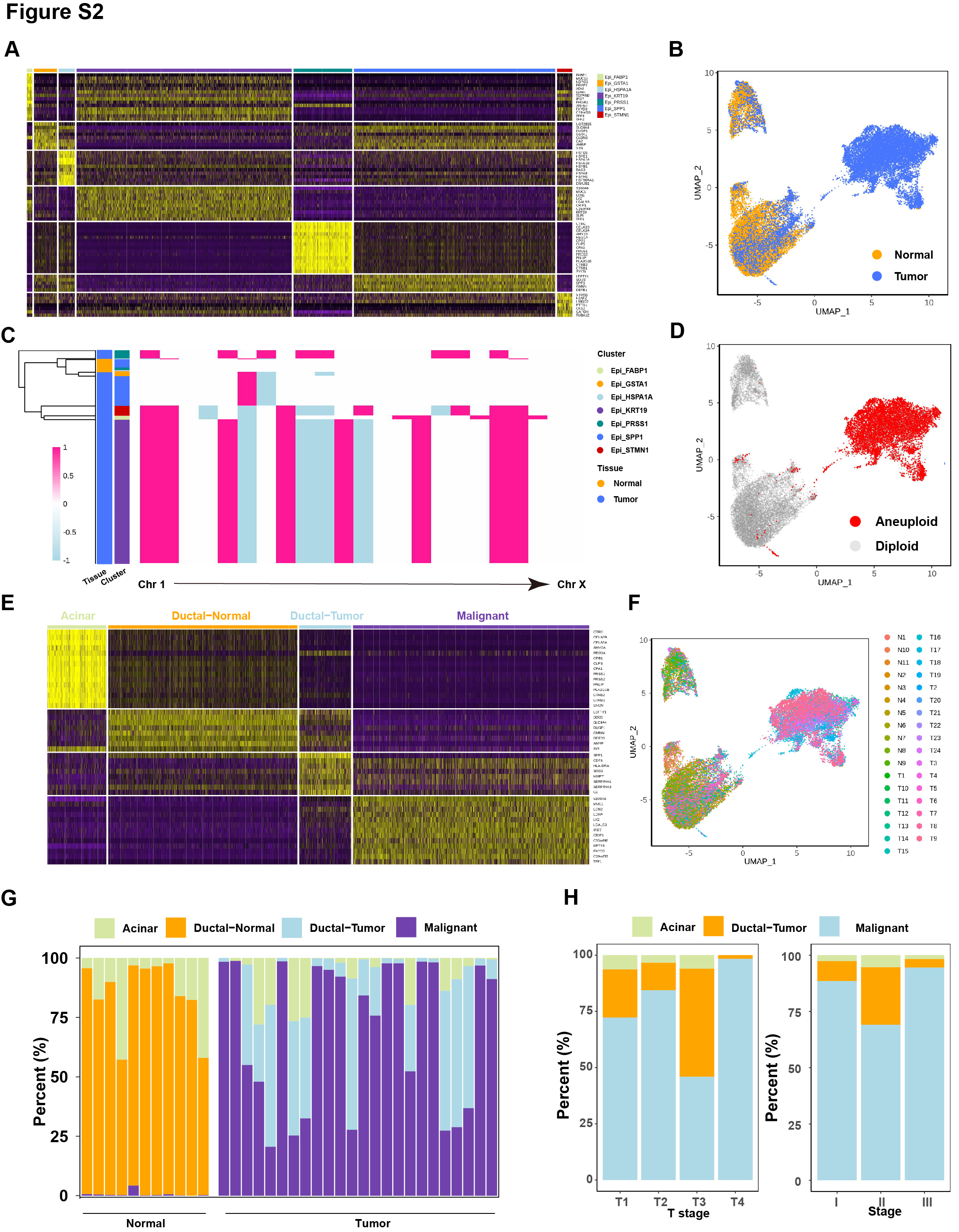

Supplement: Supplementary file 7 [file Image2.JPEG]
